# Fibrillar Self-Assembly of a Chimeric Elastin-Resilin Inspired Engineered Polypeptide

**DOI:** 10.3390/nano9111613

**Published:** 2019-11-14

**Authors:** Angelo Bracalello, Valeria Secchi, Roberta Mastrantonio, Antonietta Pepe, Tiziana Persichini, Giovanna Iucci, Brigida Bochicchio, Chiara Battocchio

**Affiliations:** 1 Department of Sciences, University of Basilicata, Via Ateneo Lucano, 10, 85100 Potenza, Italy; angelo.bracalello@unibas.it (A.B.); antonietta.pepe@unibas.it (A.P.); 2 Department of Sciences, University of Roma Tre, Via della Vasca Navale, 79, 00146 Rome, Italy; roberta.mastrantonio@uniroma3.it (R.M.); tiziana.persichini@uniroma3.it (T.P.); giovanna.iucci@uniroma3.it (G.I.)

**Keywords:** elastin, resilin, nanofibers, circular dichroism, cytotoxicity, self-assembly

## Abstract

In the field of tissue engineering, recombinant protein-based biomaterials made up of block polypeptides with tunable properties arising from the functionalities of the individual domains are appealing candidates for the construction of medical devices. In this work, we focused our attention on the preparation and structural characterization of nanofibers from a chimeric-polypeptide-containing resilin and elastin domain, designed on purpose to enhance its cell-binding ability by introducing a specific fibronectin-derived Arg-Gly-Asp (RGD) sequence. The polypeptide ability to self-assemble was investigated. The molecular and supramolecular structure was characterized by Scanning Electronic Microscopy (SEM) and Atomic Force Microscopy (AFM), circular dichroism, state-of-the-art synchrotron radiation-induced techniques X-ray photoelectron spectroscopy (XPS) and near-edge X-ray absorption fine structure spectroscopy (NEXAFS). The attained complementary results allow us to assess as H-bonds influence the morphology of the aggregates obtained after the self-assembling of the chimeric polypeptide. Finally, a preliminary investigation of the potential cytotoxicity of the polypeptide was performed by culturing human fetal foreskin fibroblast (HFFF2) for its use as biomedical device.

## 1. Introduction

In the research framework of tissue engineering, recombinant protein-based biomaterials are promising candidates for the construction of medical devices. The biopolymers are composed of block polypeptides combined by ligation of genes encoding polypeptide sequences inspired by different proteins with tunable properties conferred by each block domain. The inspiring models are mainly natural proteins, conferring to tissues and organs the properties of elasticity and strength. The polypeptide is a sort of chimera. The chimera is a mythical hybrid monster composed of the parts of more than one animal: a lion with the tail of a snake and the head of a goat. Analogously, the chimera could be made of different proteins, such as elastin, resilin, and collagen (REC)-inspired sequences [1]. REC exhibited tunable self-assembling and a quasi-ideal elastomeric behavior [2]. In general terms, elastin-inspired sequences have been widely employed both in structural studies aimed to elucidate the mechanism of elasticity of the protein and in recombinant engineered polypeptides [3,4], while resilin-like polypeptides were less investigated [5]. Nevertheless, resilin protein, found in insects like dragonflies and cicadas, exhibits outstanding elasticity and resilience properties with Young’s modulus of 50–300 kPa and a resilience value greater than 92% [6,7]. Furthermore, resilin is less hydrophobic than elastin, conferring higher solubility to the polypeptide in aqueous solution. In 2005, Elvin produced for the first time the recombinant pro-resilin, rec1-resilin. Rec1-resilin from the N-terminal elastic repeat domain (Exon 1) of the *D. melanogaster CG15920* gene comprised 18 repeats of a 15 amino acid sequence: GGRPSDSYGAPGGGN [6]. Subsequently, Exon 1 and 3 of the native *D. melanogaster* CG15920 gene were cloned and expressed [8]. Kiick and co-workers have synthesized a polypeptide constituting 12 repeats of the resilin putative consensus sequence GGRPSDSFGAPGGGN encoded by the first exon of *D. melanogaster* [9]. Resilin-like peptides integrated with bioactive sequences were also expressed [10]. Furthermore, Liu and co-workers used 10 and 30 repeats of the *A. gambiae* consensus sequence AQTPSSQYGAP with Y replaced by F and lysine (K) [11,12]. A recombinant resilin/elastin-like diblock copolypeptide having sequences different from those used in the present work were produced in a recent paper from Chilkoti and co-workers [13,14]. The diblock copolypeptides exhibited both lower and upper critical solution temperature phase behavior according to the length of the resilin- or elastin-mimetic blocks and self-assembled into spherical or cylindrical micelles.

Herein, we present the design, preparation, and structural investigation of a chimeric polypeptide made of sequences inspired by resilin and elastin (RE). It is worth noting that, although data on the supramolecular structures of natural resilin fibers are lacking, elastin is present as fibers in tissues, conferring them elasticity [15]. Therefore, the assessment of the propensity to self-assemble in fibers by RE polypeptide is of outstanding interest in the perspective of its use as a potential elastic biomaterial. This work represents an original example of the study of a sequence-defined, monodisperse diblock polypeptide of hydrophilic resilin- and hydrophobic elastin-like polypeptide blocks that undergoes self-assembly, demonstrated by UV-spectroscopy, in fibers. The morphology of the fibers were investigated by Scanning Electron Microscopy (SEM) and Atomic Force Microscopy (AFM), while the molecular and supramolecular structure of the polypeptide fibers were widely characterized by means of circular dichroism (CD) and complementary state-of-the-art synchrotron radiation (SR)-induced spectroscopic techniques, namely X-ray photoelectron spectroscopy (SR-XPS) and near-edge X-ray absorption fine structure spectroscopy (NEXAFS). Finally, the preliminary biological studies have ascertained its ability to interact with human fetal foreskin fibroblast (HFFF2).

## 2. Materials and Methods 

### 2.1. Materials

#### 2.1.1. Production of *rel* and *eln* DNA Genes

The DNA sequences for *rel* was designed, synthesized, and cloned in p10 vector (Italian Patent N.0001429709). DNA sequence for *eln* monomeric gene with optimized codon for expression in *Escherichia coli* was synthesized and cloned in pUC57 vector (Invitrogen) by GenScript. DNA sequences are shown in Figure S1.

#### 2.1.2. Construction of Re (Rel + Eln) Gene

Recombinant DNA of p10 and pUC57 vectors were used as templates in Polymerase chain reactions PCR for the production of *rel* and *eln* DNA fragments, respectively. Details on primers, PCR cycles and materials are indicated in Table S1. The PCR products were separated on a 1.2% agarose gel (Sigma-Aldrich, Milan, Italy) in TBE Buffer (90 mM of Tris-Borate; 2 mM of EDTA), carrying out electrophoresis at 110 V. The bands containing the *rel* and *eln* DNA fragments were excised from the gel, purified using the NucleoSpin Gel, and PCR Clean-Up (Macherey-Nagel GmbH & Co, Düren Germany). Finally, the *rel* and *eln* DNA fragments were inserted into pDrive vectors (Qiagen Srl, Milan Italy) and transformed in DH5α competent *E. coli* cells. Clones were selected for resistance to ampicillin. Correct constructs were verified by DNA sequence analysis (Eurofins Genomics, Ebersberg, Germany). All sequences were generated by using BigDye™ Terminator v3.1 Cycle Sequencing Kit (Thermo Fisher Scientific, Waltham, MA USA) following standard protocols. For sequencing reactions, peqStar 96 High Pressure Lid HPL (PEQLAB Biotechnologie GmbH, Erlangen, Germany), GeneTouch (Biozym Scientific GmbH, Oldendorf, Germany), or Biometra TAdvanced (Analytik Jena, Jena, Germany) thermal cyclers were used. Sequencing reaction cleanup was done either manually or on a Hamilton Starlet robotic workstation (Hamilton Robotics GmbH, Martinsried, Germany) by gel-filtration through a hydrated Sephadex matrix filled into appropriate 96-well filter plates, followed by a subsequent centrifugation step. Finally, all reactions were run on ABI3730xl capillary sequencers equipped with 50 cm capillaries and POP7 polymer (Thermo Fisher Scientific, Waltham, MA USA). Sequencing data was generated by using the original ABI Software, including the KBbasecaller (Thermo Fisher Scientific, Waltham, MA, USA). The pDrive + *rel* plasmid was digested with BbsI restriction endonuclease and treated with CIP (calf intestinal alkaline phosphatase). The pDrive + *eln* vector was digested with BbsI, and the products were separated on a 1.2% agarose gel and purified as described above. *Eln* sequence contains the restriction site for BbsI at 3’ end and is shown in Table S1, where the restriction site is underlined. The BbsI/BbsI *eln* fragment and the linearized pDrive + *rel* vector were ligated by 2 h incubation at 16 °C, in the presence of T4 DNA Ligase (Biolabs, Ipswich, MA, USA). Colonies were screened on LB agarose plates supplemented with 100 μg/mL ampicillin, and the correct construct (734 bp) was verified by DNA sequence analysis of recombinant plasmid isolated from a single transformant.

#### 2.1.3. Expression and Purification of the Recombinant RE Polypeptide

The pDrive + *re* vector, obtained from the selected colonies, was used as the template for PCR reaction, using the primers described in Table S1. The PCR product was cloned into pET46EK/Lic vector (Novagen, Merk Life Science S.r.l, Milan, Italy), containing a strong T7Lac promoter and an amino terminal His-tag coding sequence, immediately followed by an EK/Lic cloning site. The recombinant plasmid was used to transform BL21star(DE3) competent *E. coli* cells (Novagen, Merk Life Science S.r.l, Milan, Italy), and clones were selected for resistance to ampicillin. After cloning into the expression plasmid, the sequence of the chimeric gene was confirmed by DNA sequencing as previously described.

Bacterial culture was grown at 37 °C overnight in LB medium supplemented with 100 μg/mL of ampicillin and subsequently used to inoculate 1 L of LB medium containing the antibiotic ampicillin 100 μg/mL and incubated at 37 °C, with shaking until the OD_600_ was 0.6–0.8.

Gene expression was induced by the addition of 0.5 mM of IPTG (isopropyl-β-D-thiogalactopyranoside) for 1 h at 37 °C. BL21star(DE3) *E. coli* cells containing the pDrive + *re* vector were then harvested by centrifugation, resuspended in Binding Buffer (20 mM of Na_2_HPO_4_, pH 8, 10 mM of imidazole, 0.5 M of NaCl + 0.1% Tween 20), and lysed by ultrasonic disruption. To test the fusion protein expression, Western blotting, using Amersham PVDF Hybond Transfer Membrane (Euroclone SpA, Pero, Italy) and monoclonal antipolyhistidine peroxidase conjugate antibody, was performed (Figure S2).

The supernatant containing the recombinant His_6_ tag polypeptide was recovered after centrifugation at 17,500 g for 20 min at 4 °C and subsequently purified by a NiNTA agarose beads, using standard procedures (Qiagen Srl, Milan Italy). Briefly, the resin was pre-equilibrated with Binding Buffer, and, after loading of the supernatant, the mixture was shaken for 1 h. The resin was washed with Binding Buffer, and the His_6_ tag polypeptide was eluted from the resin, using Elution Buffer (20 mM of Na_2_HPO_4_, pH 8, 500 mM of imidazole, 0.5 M of NaCl). This protocol yielded about 35–40 mg of RE polypeptide per liter of culture.

### 2.2. Methods

#### 2.2.1. Peptide Purification

RE polypeptide was dissolved in ultra-pure water in resistivity 18.2 MΩcm and inorganic reduction up to 99.99% (TOC level to <10 ppb) produced by aquaMAX TM Ultra 370 Series (Youngin Chromass, Gyeonggi-do, Korea). Then, it was frozen at −20 °C and lyophilized by Alpha 1–2 LD Plus Freeze-Dryer (Martin Christ, Osterode, Germany). The polypeptide was solubilized in 0.1% TFA in H_2_O at a final concentration of 1 mg/mL. It was then purified by semipreparative reversed-phase high-performance liquid chromatography (RP-HPLC). The instrument was a LC-20 AD (Shimadzu Italia S.r.l., Milan Italy) (equipped with SPD-M20 UV/VIS (Shimadzu Italia S.r.l., Milan Italy), and the column was a Jupiter C5 300 Å (250 × 10 mm, 5 μm) (Phenomenex, Torrance, CA, USA), and UV detection was at 220 and 280 nm. Products were eluted with a binary gradient of 0.1% TFA in H_2_O and 0.1% TFA in acetonitrile (CH_3_CN). CH_3_CN varied from 5% to 70% over 30 min, at a flow rate of 3 mL/min. The obtained chromatogram is shown in Figure S3. The collected fractions were frozen at −20 °C and lyophilized.

#### 2.2.2. Amino Acid Analysis

Identity and purity of the final product were confirmed by ESI–MS (CEINGE, Naples, Italy -Figure S4) and amino acid analysis (CiCT, Barcelona, Spain, Table S2). The amino acid analysis was carried out by dissolving the polypeptide in water (1% w/v phenol). An aliquot of α-aminobutyric acid (AABA) solution (2.5 mM) and concentrated HCl (37%) were added to obtain a final concentration of 6 M HCl. The final solution was transferred to hydrolysis tubes. Hydrolysis was performed at 110 °C for 16 h. Then, the samples were evaporated by using a rotary evaporator, resuspended in 20 mM of HCl, and filtered. Aliquots of the filtered solutions were derivatized with 6-aminoquinolyl-N-hydroxy-succinimidyl carbamate, according to the Waters AccQ-Tag method. Final analytical determination was carried out. AccQ-Tag derivatized amino acids were analyzed by HPLC with UV detection (λ = 254 nm) on a WATERS 600 HPLC gradient system equipped with a WATERS 2487 UV detector (WATERS, Milford, MA, USA.

#### 2.2.3. ESI–MS Analyses

ESI–MS analyses were performed on Waters Quattro Micro mass spectrometer (WATERS, Milford, MA, USA) equipped with a triple quadrupole and an electrospray source. The instrument was calibrated with lysozyme. Lyophilized peptides were dissolved in a mixture of H_2_O (2% CH_3_COOH) and acetonitrile (1:1 ratio). Aliquots of the solution were introduced into the electrospray ion source by a syringe pump. Spectra were elaborated by using the software provided by the manufacturer. Molecular masses are given as average values.

#### 2.2.4. CD-Spectroscopy

CD spectra of RE polypeptide (0.1 mg/mL) were acquired at different temperatures with a Jasco J-815 Spectropolarimeter (Jasco Europe, Cremella, Italy) equipped with a HAAKE thermostat as temperature controller by using 0.1 cm path length quartz cell. Samples were equilibrated at 0 °C, 25 °C, 37 °C, and 60 °C for 5 min before acquisition. Spectra were acquired by taking points every 0.1 nm, with 100 nm/min scan rate, 16 scans, an integration time of 2 s, and a 1 nm bandwidth. The data are expressed in terms of [Θ], ellipticity value, as deg·cm^2^·dmol^–1^ as a function of temperature and solvent.

#### 2.2.5. UV-Spectroscopy

Res polypeptide, produced as described in [16], and RE polypeptides (1.5 mL) at a concentration of 1 mg/mL in PBS buffer (10 mM of Na_2_HPO_4_, 1.5 M of NaCl, pH 7.0) were analyzed by UV-spectroscopy. Solutions were placed into a quartz cuvette of 1 cm path length and inserted in the sample cell of the spectrophotometer. Turbidimetry on apparent absorbance (TAA) was registered at 440 nm. The TAA value of the blank was subtracted at the starting point. Turbidity profiles were obtained for each of the polypeptides by recording the optical density as a function of time at 37 °C (Time Turbidimetry) and of temperature increased at 1 °C step slope every 2 min (Thermal Turbidimetry) from −1 °C to 44 °C for Res polypeptide and from 4 to 94 °C for RE polypeptide, with stirring at 200 rpm speed. In the latter case, the sample was cooled after scans to return turbidity to the baseline. The samples were investigated on a Cary50 UV spectrophotometer (Agilent Inc., Santa Clara, CA, USA) equipped with a Peltier temperature controller. The TAA of RE slowly increased as a function of time and was monitored until a value of 2.25 O.D. was reached. TAA values higher than 2 O.D. are not significant, considering logarithmic increment of TAA values and the limitations of the spectrophotometer to ensure linearity above this value. The transition temperature (T_t_) was calculated as the temperature value where the TAA reaches 50% between maximum and minimum. At the end of the turbidity assay, the obtained suspension was centrifuged. The supernatant was removed from the pellet. Five microliters of the suspension were deposited on silicon (100) wafer substrates (Aldrich, Saint Louis, MO, USA). The samples were air-dried and repeatedly rinsed with ultra-pure water, in order to remove salts, and then analyzed by using AFM and SEM microscopies.

#### 2.2.6. AFM

The AFM images were observed by using an XE-120 microscope (Park Systems) in air and at room temperature. Data acquisition was carried out in intermittent contact mode, at scan rates between 0.4 and 3 Hz, using rectangular Si cantilevers (NCHR, Park Systems, Suwon, Korea) with a radius of curvature less than 10 nm and with the nominal resonance frequency and force constant of 330 kHz and 42 N·m^–1^, respectively. Fiber width measurements were carried out on each micrography by using the manual method by ImageJ (U.S. National Institutes of Health, Bethesda, MD, USA). Diameter measurements are shown in Figure S5 as an example. 

#### 2.2.7. SEM

The morphology of RE aggregates was observed by using a scanning electron microscope (ESEM XL30-LaB_6,_ Philips-Fei, Hillsboro, OR, USA). Samples for SEM analysis, prepared as indicated in Section 2.2.5, were mounted using carbon tape on aluminum SEM stubs. Coating of the samples with a 5–10 nm gold layer was performed by the sputter coater Emitech K950 (Quorum Tech., Lewes, UK) using a current of 15 mA and an Argon pressure of 0.05 Torr for 2 min. SEM images were acquired with a voltage of 20 kV at different magnifications. Fiber width and morphology analysis was performed by ImageJ (U. S. National Institutes of Health, Bethesda, Maryland, USA).

#### 2.2.8. Sample Preparation of RE Polypeptide in Aggregated State

To obtain RE polypeptide in the aggregated state lyophilized RE were solubilized in PBS (10 mM, pH 7) at a final concentration of 2 mg/mL and kept at 37 °C under magnetic stirring at 200 rpm for 20 days.

#### 2.2.9. X-ray Photoelectron Spectroscopy

Synchrotron radiation (SR)-induced XPS measurements were performed at the materials science beamline (MSB) at the Elettra synchrotron radiation source (Trieste, Italy). MSB, placed at the left end of the bending magnet 6.1, is equipped with a plane grating monochromator that provides light in the energy range of 21–1000 eV. The UHV endstation, with a base pressure of 2 × 10^−10^ mbar, is equipped with a SPECS PHOIBOS 150 hemispherical electron analyzer, low-energy electron diffraction optics, a dual-anode Mg/Al X-ray source, an ion gun, and a sample manipulator with a K-type thermocouple attached to the rear side of the sample. Photoelectrons emitted by C1s, O1s and N 1s core levels were detected at normal emission geometry using photon energy of 630 eV impinging at 60°. Energy resolution of binding energies (BEs) are reported after correction for charging using the aliphatic C1s as a reference (BE 285.0 eV) [17]. Core level spectra were fitted with a Shirley background and Gaussian peak functions [18,19].

#### 2.2.10. Near Edge X-ray Fine Structure (NEXAFS) Spectroscopy

NEXAFS measurements were performed at the materials science beamline (MSB) at the Elettra synchrotron radiation source (Trieste, Italy). The nitrogen K-edge spectra were collected at normal (90°), magic (54.7°), and grazing (20°) incidence angles of the linearly polarized photon beam, with respect to the sample surface using the nitrogen KVV Auger yield. The energy resolution for the N K edge spectra was estimated to be 0.35 eV. The raw NEXAFS data were normalized to the intensity of the photon beam, measured by means of a high-transmission gold mesh and divided by corresponding spectra of the clean sample, recorded under identical conditions. To prepare RE and aggregated RE samples for the SR-XPS and NEXAFS studies, Au/Si (111) substrates surfaces were covered with 100 μL of RE solutions (2 mg/mL in PBS) or with 100 μL of RE aggregated suspensions (Section 2.2.8) dried overnight, and then rinsed under Ar flux.

### 2.3. Biological Studies

#### 2.3.1. Materials for Biological Characterization

Dulbecco’s modified Eagle’s medium (DMEM), fetal bovine serum (FBS), 0.25% Trypsin–EDTA solution, gentamicin solution 50 mg/mL, and an MTT assay kit were obtained from Sigma-Aldrich (Milan, Italy).

#### 2.3.2. Cell Cultures

Human fetal foreskin fibroblast (HFFF2) was routinely cultured in DMEM medium supplemented with 10% FBS, 40 ug/mL of Gentamicin, and 2 mM of L-glutamine, at 37 °C, in a humidified 5% CO_2_ incubator.

#### 2.3.3. Treatments

For the experiments to test cell viability, RE was resuspended in Milli-Q water at 1 mg/mL concentration and left at room temperature until use. Cell cultures were treated with 300 μL of RE in 1 mL of culture medium or with 300 μL of H_2_O as control.

For the experiments to analyze whether RE could promote cell adhesion, we first prepared RE nanofibers. To this aim, lyophilized RE was resuspended in phosphate buffer at final concentration of 2 mg/mL and then incubated at 37 °C, under stirring, for 20 days. Before each experiment, sterilization was achieved by exposing samples to UV radiation for 6 h. To exclude any structural damage, the stability of the oligopeptide chemical structure was checked by FT-IR measurements before and after sterilization.

#### 2.3.4. MTT Assay

HFFF2 were seeded in 24-well plates, at a concentration of 20 × 10^3^ cells/well. Cells were treated with RE (300 μL) or H_2_O (300 μL) diluted in 1 mL final volume of culture medium or in 1 mL of undiluted culture medium used as positive control, and they were allowed to grow for 24, 48, and 72 h. Then, at the end of the incubation periods, the viability of HFFF2 cells was analyzed by MTT assay, as indicated by the manufacturer’s instructions. Briefly, MTT solution (stock solution of 0.5 mg/mL) was added to cell cultures at the final concentration of 10%. After incubation at 37 °C for 4 h, formazan crystals were dissolved in lysis buffer (4 mM of HCl, 0.1% NP40 (v/v) in isopropanol) and the optical density (O.D.) of each sample was measured by using a microplate reader at 570 nm (BioTek 28 ELx800 Absorbance Microplate Reader, Winooski, VT, USA).

Cell viability of HFFF2 treated with RE was calculated relative to the viability of HFFF2 cells used as control.

#### 2.3.5. Evaluation of Living and Dead Cells by Trypan Blue Staining

In order to evaluate cells adhesion, 5 × 10^4^ HFFF2 were seeded on ultra-low adhesion multi-well plates coated with (aggregated) RE nanofibers. To this aim, 1 mL suspension of RE nanofibers (300 μL/mL in H_2_O) was added to the plates and left to dry overnight, at room temperature, under sterile conditions. HFFF2 seeded on standard tissue culture plates were used as positive control, whereas H_2_O coated or phosphate buffer-coated ultra-low adhesion plates were considered as negative control. After 24 and 72 h from seeding, cells were detached by trypsin-EDTA and counted, using a Neubauer chamber. In order to distinguish dead cells, HFFF2 was stained with Trypan Blue. All experiments were repeated twice.

#### 2.3.6. Statistical Analysis

Statistical analysis was carried out by using the analysis of variance (ANOVA, followed by Bonferroni test). A *p*-value of <0.05 was accepted as indicative of a statistically significant difference.

## 3. Results and Discussion

### 3.1. Resilin-Elastin (RE) Polypeptide Design

**Figure 1:**
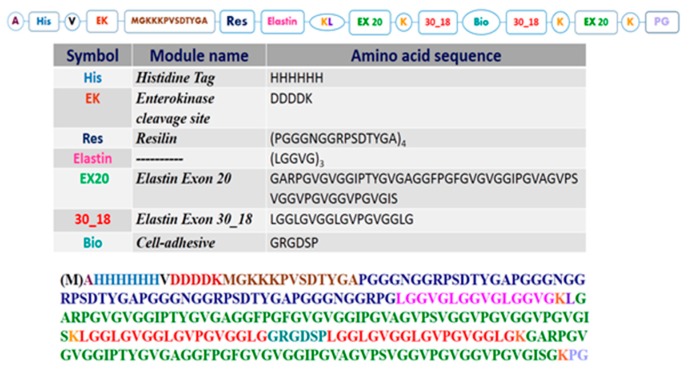
Cartoon schematizing the primary structure of resilin–elastin polypeptide (RE).

A schematic representation concerning the blocks of RE polypeptide is shown in Figure 1.

Elastin blocks are made up of elastin portion containing sequences encoded by exon 20 (EX20- aa 383–438 of UniProtKB entry P15502) and exon 30 (EX30_18, aa 709-726 of UniProtKB entry P15502) of human tropoelastin gene (HTE) and glycine-rich repeats (LGGVG) motif as element of novelty in comparison to REC polypeptide, where only (LGGVG) was present [20]. The rationale for the introduction of EX30_18 and EX20 in the chimeric polypeptide is due to their property to undergo coacervation [21,22]. The coacervation is a physiological process characteristic of elastin and essential in elastic fiber formation [23]. It consists in a phase separation that occurs on heating the full protein in solution. During this process, the increase of the temperature causes the solution to become cloudy with separation of a dense viscoelastic phase from a lighter aqueous phase. The process is fully reversible: the cloudiness disappears when the solution cools down. The coacervates were observed by transmission electron microscopy, showing that isolated EX20 and EX30_18 peptides form huge bundles of fibers exhibiting structural organization similar to that of natural elastic fibers in thin sections of tissues [24,25,26]. Furthermore, to improve the interaction of the chimeric polypeptide with cells, the cell-binding RGD motif from fibronectin [27,28,29,30] was inserted in RE polypeptide. Analogously to REC polypeptide, the resilin block was therein constituted of the repeat domain corresponding to aa sequence 108-122/123-137/175-189/190-204 of UniProtKB entry Q9V7U0, identified as pro-resilin of *D. melanogaster* [5,6,9]. 

### 3.2. Polypeptide Characterization

#### 3.2.1. Amino Acid Analysis 

Identity of the polypeptide was assessed by amino acid analysis. The data are summarized in Table S2, where experimental and theoretical results of amino acid composition of RE are shown. The two data sets are in good agreement (<3%).

#### 3.2.2. Mass Spectrometry Analysis

RE polypeptide was purified by reverse-phase HPLC. The chromatogram is shown in Figure S3. The main fractions were collected at the following retention times: 16.2, 18.6, and 22.4 min, labeled as fraction 1, 2, and 3, respectively. The collected fractions were analyzed by electrospray mass spectrometry, in order to assess the molecular weight. The analysis showed that fraction 3 in Figure S3 contained RE polypeptide, while the others contained polypeptides of smaller size. ESI–MS analysis of fraction 3 is shown in Figure S4 and evidenced a dominant peak at 22,443 Da, assigned to the average mass of RE polypeptide as protonated species [M+H]^+^ and calculated with an isotopident tool [31].

The value corresponds to the primary structure of RE polypeptide devoid of N-terminal methionine residue (Figure 1). The loss is due to cleavage by the enzyme methionine aminopeptidase (MAP), frequently encountered in recombinant proteins expressed in bacterial expression systems when the amino acid following N-terminal methionine is of small size [32]. A minor peak at 22,500 Da is assigned to the presence of salt adduct [M +(n−2)H+Na+K]^n+^. 

### 3.3. Circular Dichroism

**Figure 2:**
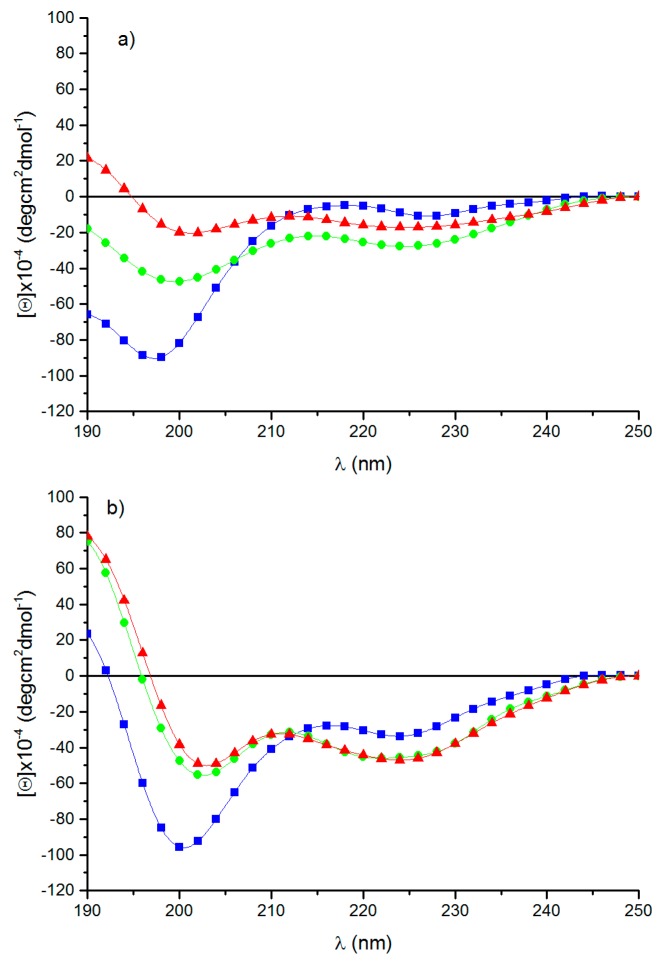
CD spectra of RE polypeptide at 0°C (■), 37°C (●), and 60 °C (▲) in (**a**) aqueous solution and (**b**) TFE.

Circular dichroism is a useful and quick tool for investigating the secondary structure of proteins and peptides. Figure 2 shows the spectra of RE polypeptide recorded as a function of temperature and solvent. In aqueous solution at 0 °C, the spectrum shows a strong negative band centered at 198 nm, together with two negative peaks having a tendency to adopt positive and negative values at about 218 and 227 nm, respectively (Figure 2a).

The spectrum is indicative of the presence of poly-L-proline II left-handed helix together with unordered conformations [33]. The increase of the temperature to 37°C and 60°C induces the progressive increment of the bands at 198 and 227 nm, which, at 60 °C, appears slightly blue-shifted to 225 nm [34]. Additionally, at 60 °C, a positive band appears at about 190 nm, suggesting the presence of type I β-turn conformations. Since 2,2,2-trifluoroethanol (TFE) is well-known for favoring folded conformations as helices and β-turns promoting intramolecular hydrogen bonding [35,36,37], the measurements of RE polypeptide spectra were carried out in TFE (Figure 2b). At a low temperature, the spectrum shows a strong negative band at 200 nm and a weaker negative band at 225 nm. The spectrum dramatically changes when the temperature reaches 37°C and 60 °C. A positive band at about 190 nm, with two weak negative bands at 202 and 224 nm, are the spectral features of the spectra. The increase to 60 °C does not change the spectrum. The spectra are diagnostic of type I-III β-turn stable at high temperatures [38]. The increase of the stability of turn conformations on warming was previously found for resilin/elastin-like peptides [39,40]. CD spectra of RE polypeptide demonstrate the presence of different conformations as PPII, random coil and turns quickly, interconverting among them [34]. The conformational studies were investigated as a function of two variables, i.e., temperature and solvent. Previous studies have demonstrated the influence of the temperature on specific conformations as, for example, PPII, which is stable at a low temperature. The change of conformation with the temperature evidenced the flexibility of the polypeptide chain. Furthermore, solvent polarity affected the conformation, too, as shown in the spectra carried out both in aqueous solution and in TFE, well-known for triggering the adoption of different secondary structures [34]. 

### 3.4. Self-Aggregation

**Figure 3:**
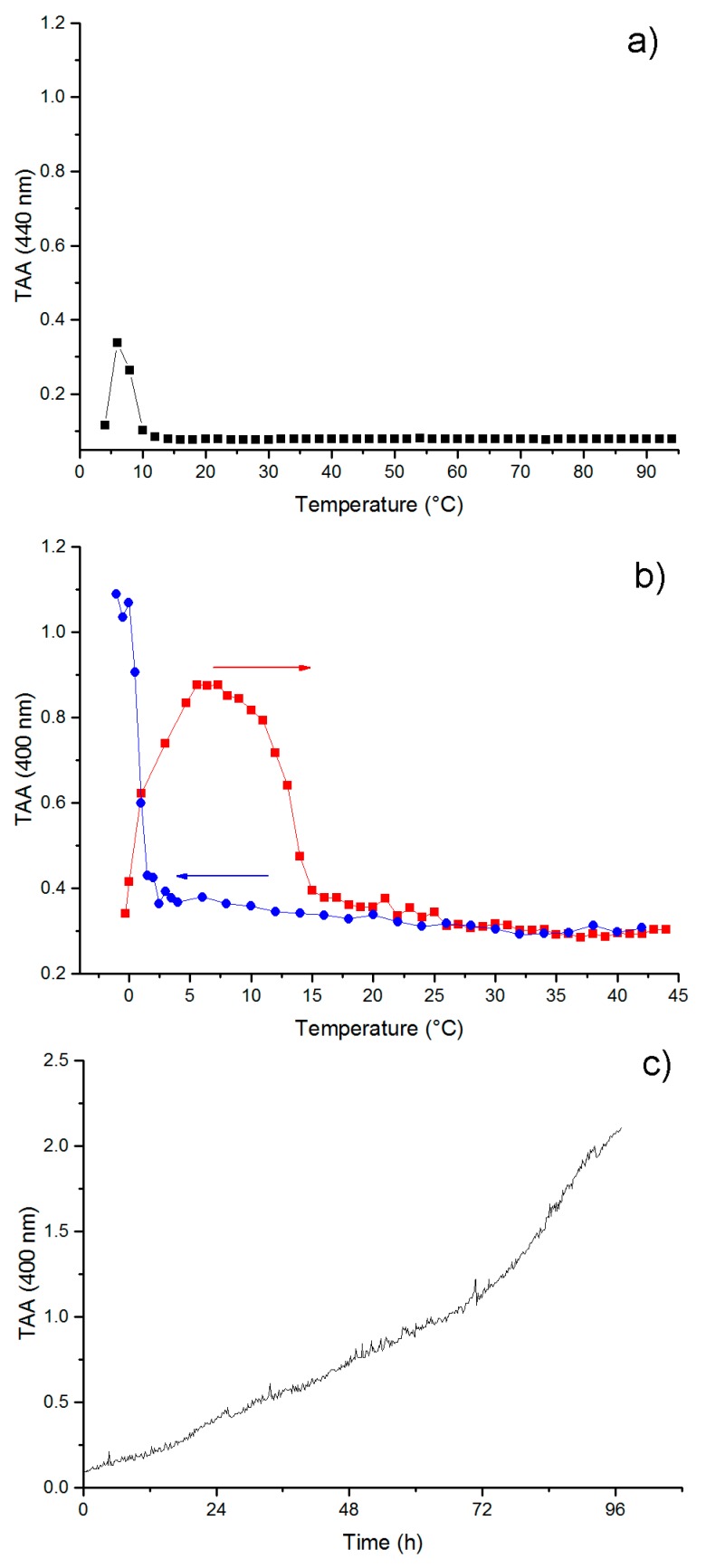
Turbidimetry assay carried out on (**a**) RE (1 mg/mL) in PBS as a function of temperature; (**b**) Res (1 mg/mL) in PBS as a function of temperature; (**c**) RE (1 mg/mL) in PBS at 37 °C as a function of time.

Self-aggregation of RE polypeptide was monitored by turbidimetry assay, registering the attenuation of the incident beam by light scattering due to the presence of aggregates. For turbidity measurements, we employed a UV-spectrophotometer, recording the apparent absorbance at 400 nm as a function of temperature or time. In Figure 3a, the turbidity of RE polypeptide in PBS was monitored as a function of temperature. A low-temperature aggregation phase transition was observed as shown by the increase of the absorbance at 6 °C. A further increase of the temperature dissolved the aggregates, as very low absorbance values were registered on increasing the temperature to 95 °C. The low-temperature aggregation can be ascribed to cold coacervation, a phase separation previously described for resilin-derived polypeptides [10]. In order to confirm the propensity of the resilin sequence inserted in the RE polypeptide to undergo reversible upper critical solution temperature (UCST) phase transition, we performed a turbidimetry assay of the Res polypeptide in PBS as a function of temperature. In Figure 3b, the warming curve and the cooling curve of the TAA measurements were shown, highlighting for resilin block (Res) a reversible UCST transition at 1 °C (Figure 3b). 

Previous studies carried out on resilin-inspired peptides highlighted the propensity to self-assemble into fibrillar structures after 12 h of incubation at room temperature [16]. Accordingly, we performed turbidimetry measurements of RE polypeptide at 37 °C as a function of time. As shown in Figure 3c, a gradual increase of the apparent absorbance was observed for RE polypeptide, which reached values above 2 O.D. 

**Figure 4:**
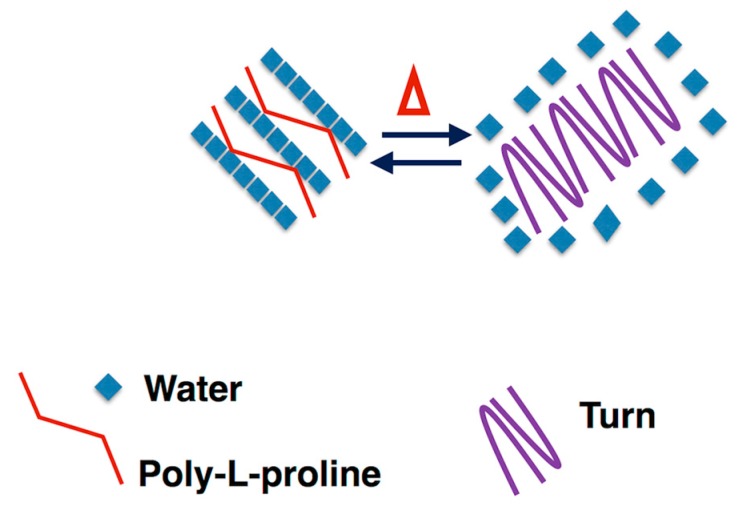
Cartoon of the proposed self-aggregation mechanism of RE polypeptide. Cold coacervation is triggered by extended and flexible conformations as PPII and random coil, while folded turns favor the self-aggregation at 37 °C by expelling water molecules.

Lyons and co-workers showed that an aqueous solution of a recombinant resilin polypeptide displays UCST at 4 °C, leading to a separation into a protein-rich phase and called cold-coacervation [10]. Concerning the elastin block of the chimeric RE polypeptide, and the sequences inspired by EX20, EX30_18, and (LGGVG)_3_, it is well-known that all of them are able to give rise to fibers [20,21]. On that basis, the chimeric polypeptide is expected to form fibers, as well. However, while EX20 and EX30_18 are able to coacervate as elastin does by forming elastin-like bundles of fibers, previous work has demonstrated that polypeptide sequences containing the consensus repeating sequence (XGGZG)_n_ with V and/or L as guest residues are able to give rise to amyloid-like fibers [21]. In other words, the coexistence in RE polypeptide sequence of elastin-blocks able to reversibly self-aggregate in a coacervate, as EX20 and EX30_18, together with the amyloidogenic sequence (LGGVG)_3_, does not allow us an early preview of the final aggregation state of RE polypeptide. RE polypeptide was able to give rise to a cold coacervation, as expected for resilin-inspired polypeptides. However, the coacervation at higher temperatures, typical of elastin, was not observed in thermal turbidimetry of RE polypeptide. In fact, the self-aggregation occurred at 37 °C, very slowly, and in a gradual, noncooperative way (Figure 3c). Generally, tropoelastin and elastin-like peptides undergo a conformational transition toward folded turns before the coacervation [23,41]. Interestingly, CD spectra show that, while at 0 °C, the RE polypeptide assumes extended and flexible conformations as PPII, and at 37 °C, RE polypeptide adopts more folded conformations as turns (Figure 2). In summarizing, to the best of our knowledge, this work demonstrates for the first time that RE polypeptide is able to keep the self-aggregation characteristics of the constituent proteins. Even if in a speculative way, we can infer that cold coacervation is triggered by extended and flexible conformations as PPII and random coil, while folded turns favor the self-aggregation at 37 °C. At a low temperature, water molecules act as a plasticizer on extended polypeptide chains fixed by slow-chain kinetics and appearing almost frozen in elongated shapes. We assume the presence of highly ordered water molecules, sort of like clathrates, interposed among the single polypeptide chains that appear very close to each other. The working model at high temperatures, instead, infers that folded turns expel water molecules. In other words, the driving force for the self-aggregation mechanism could be the hydrophobic force generated by the interaction of polypeptide chains (Figure 4).

### 3.5. Morphological Characterization

**Figure 5:**
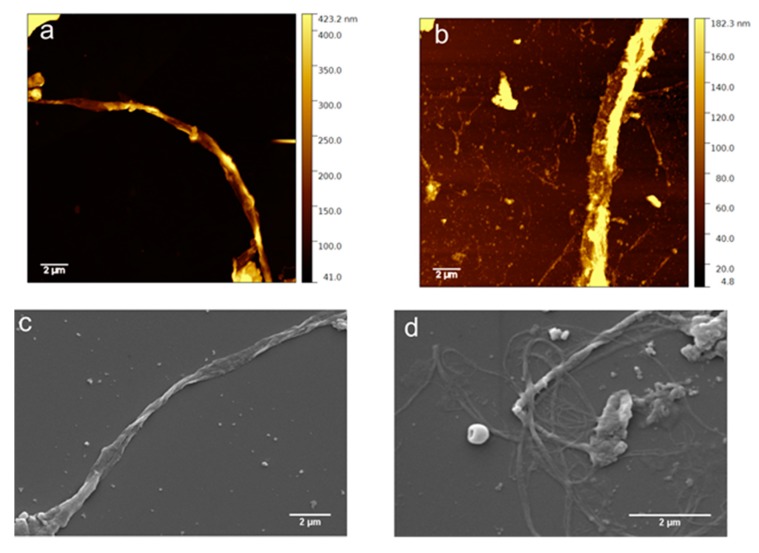
Aggregated RE polypeptide: (**a**,**b**) AFM; (**c**,**d**) SEM.

The morphology of the aggregates formed by RE polypeptide has been observed by microscopy techniques (Figure 5). AFM and SEM images show long fibers of not uniform diameter along the fiber. AFM images show a long fiber with a diameter ranging from 0.28 to 0.91 μm (Figure 5a; Figure S5) and from 1.67 to 1.87 μm (Figure 5b). SEM images exhibit fibers whose diameter is in the range 0.37–1.06 μm (Figure 5, panel c). Fibers with diameters in the range 0.24–0.67 μm organized in bundles are also shown (Figure 5d). The thinner diameters observed by SEM are ascribed to the dehydration effect caused by high vacuum conditions (10^−3^ Pa) present in the sample chamber of SEM. In this condition, water is strongly out of equilibrium, evaporates, and is pumped out very quickly. Overall, the results show that the resilin/elastin-inspired polypeptide gave rise to fibers similar to those reported in the literature for the entire elastin protein [23,24,25]. In the absence of data on the supramolecular structures from natural resilin, we could consider the supramolecular structures of each block composing RE polypeptide. A previous study carried out on the synthetic esaconta resilin-inspired peptide (Res), the resilin block present in RE, demonstrated a strong tendency to form fibril structures after 12 h of incubation at room temperature. The elastin-like motif (LGGVG)_3_ self-aggregated in helical and unbranched structures typical of amyloid-like fibers [21] differently from EX20 and EX30_18 sequences giving rise to bundles of intertwined fibers [22,41]. The result on the morphology of RE aggregated structures show that it is not amyloid-like, as observed by SEM and AFM microscopies. Nevertheless, the morphology of the fibers is analogous to that observed for elastin protein and elastin-derived sequences and very different from amyloid-like fibers [30]. These findings are noteworthy because, to the best of our knowledge, no previous studies evidenced the formation of fibers in a resilin–elastin polypeptide. Chilkoti and co-workers published a resilin/elastin diblock copolypeptides exhibiting LCST and UCST phase behavior and able to self-assemble into spherical or cylindrical micelles, depending on the resilin/elastin ratio that was rationalized according to simple polymer physics principles [13].

The present results addressing the presence of fibers by RE polypeptide is a hallmark in the design of innovative biomaterials because elastin natural protein exert its biological function, elastic function, in the form of fibers.

### 3.6. Chemical and Electronic Structure Characterization

High-resolution X-ray photoelectron spectroscopy induced by synchrotron radiation (SR-XPS) gave detailed information on the chemical composition and the electronic interactions among different atoms, either strong, as covalent or ionic bonds, or weak, as H-bonds [17]. SR-XPS investigations were performed on both lyophilized (i.e., not aggregated) and aggregated RE polypeptide. The scope was the comparison of their electronic, molecular, and supramolecular organization. All samples were deposited on polycrystalline gold surfaces, as described in the experimental method (Section 2.2.10). SR-XPS measurements were carried out at the C1s, N1s, and O1s core levels for all samples. All binding energy (B.E.), full-width half maxima (FWHM), experimental atomic ratio values, and assignments are reported in Table S3 in the Supporting Information. 

**Figure 6:**
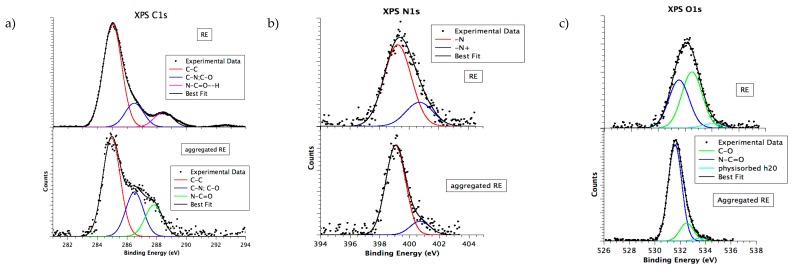
C1s (**a**), N1s (**b**), and O1s (**c**) SR-XPS spectra collected on lyophilized RE and aggregated RE. Spectral components are also reported as colored curves.

C1s spectra measured for RE polypeptide, in both lyophilized and aggregated form, are shown in Figure 6a. The C1s signal of all samples can be resolved by curve-fitting analysis into three main components corresponding to aliphatic C–C (BE = 285.0 eV, also used for all spectra BE calibration), C–N, C–O (286.5 eV) and to carbon atoms of the polypeptide backbone, respectively. C1s signal associated to N–C=O groups is found at 287.8 eV, as expected for aggregated state [18]. The signal is shifted at higher BE values (288.7 eV) for the lyophilized state. The observed chemical shift is attributed to N–C=O–H partially involved in H-bonds [42] specific for elastin-like peptides [43]. In fact, although H-bonds do not cause a change in the formal oxidation state of elements, they can greatly affect the core-level binding energy values of the involved heteroatoms [44].

N1s spectra, in Figure 6b, show two peaks corresponding to amide nitrogen of the polypeptide (BE = 399.0 eV) and protonated amine-like nitrogen (BE = 400.5 eV), as expected [45,46]. The data confirm the stability of the polypeptide primary structure upon aggregation and under the X-ray beam. It is noteworthy that N1s spectrum of the lyophilized peptide is broader than the same signal measured for the aggregated form, coherently with the presence of H-bonds suggested by the C1s N–C=O component shift [42].

O1s spectra of all samples (Figure 6c) show the presence of three Gaussian components. They are attributed to the peptide bond (B.E. 531.6 eV), to carbon single-bonded to oxygen (B.E. 532.4 eV), and to physisorbed water on sample surface (B.E. 534.0 eV). It is noteworthy that the O1s signal of aggregated RE contains a strong contribution arising by the phosphate buffer that is partially superimposed to the peptide bond signal [43], enhancing its intensity with respect to the other spectral components.

**Figure 7:**
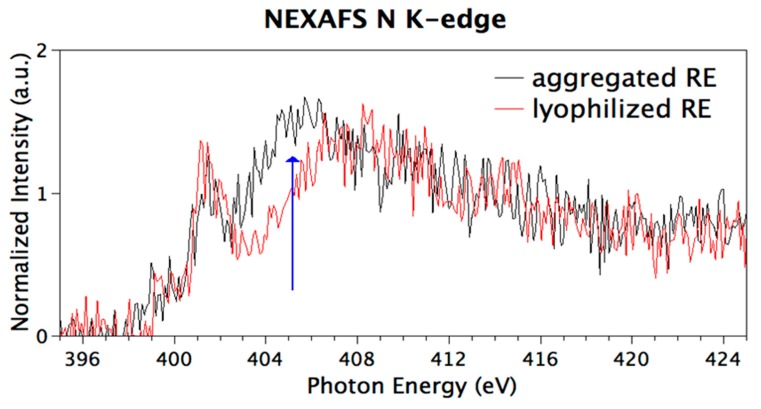
NEXAFS N K-edge spectra of lyophilized and aggregated RE polypeptide collected at Magic Incidence.

Congruently with SR-XPS data analysis, N K-edge NEXAFS spectra show resonances indicative of the functional groups expected for the specific polypeptide primary structure. Figure 7 shows N K-edge spectra collected at magic incidence (54.7° of incidence of the X-ray radiation on the sample surface, ensuring no dichroic contributions arising by sample spatial orientation) on the two samples. The sharp peak at 402 eV is assigned to N1s → π* transitions of the peptide bonds and the broad bands at 405 and 415 eV to N1s → σ* N-H and N1s → σ* N–C resonances, respectively [47]. It is well-known, in regard to self-assembled nanostructures, that N1s NEXAFS spectra are a powerful tool to investigate directional intermolecular interactions as H-bonds. Indeed, the NEXAFS resonance corresponding to the σ* N–H transition is identified as a fingerprint of the interacting state of the amine with adjacent molecules [48]. The two spectra reported in Figure 7 appear identical but for the feature related with the N1s → σ* N–H transition (blue arrow in Figure 7) that is quenched in the lyophilized RE spectrum. An analogous quenching was reported for calculated and experimental N K-edge NEXAFS spectra of small molecules having amine groups involved in H-bonds, as for example p-diaminobenzene [47] and melamine [43].

Previous studies on amyloidogenic elastin-like peptides have shown that H-bonds increase from lyophilized to aggregated samples [40]. Herein, hydrogen-bond-induced effects on SR-XPS and NEXAFS spectra are observed mainly in the lyophilized samples, suggesting that the aggregation state is not of amyloid-like nature characterized by a dense network of hydrogen bonding [49]. Even if in a speculative way, we can infer on the crucial role played by water in the amyloidogenic self-aggregation process, where it served as a bridge connecting multiple polypeptide chains [42]. Water molecules, few in the lyophilized state, are not enough for bridging polypeptide chains. Therefore, the observed hydrogen bonding occurs among backbones, differently from amyloid-like elastin-inspired peptides model. The speculation is corroborated by experimental CD studies, that demonstrated the presence of β-turns excluding the presence of β-sheet conformations considered a hallmark of amyloid-like structures [50].

### 3.7. Biological Characterization

#### 3.7.1. Cell Viability Evaluation

**Figure 8:**
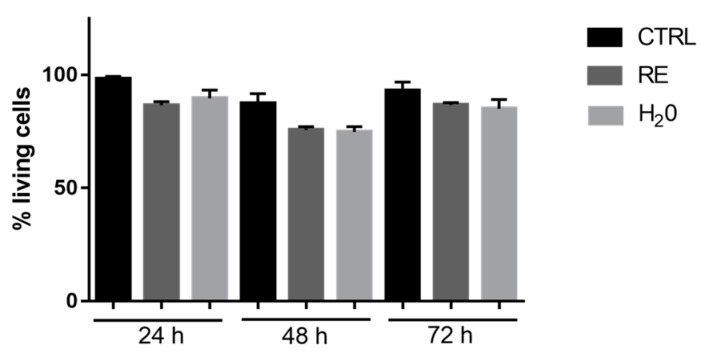
MTT assay of HFFF2 treated with H_2_O and RE (300 μL/mL).

In order to assess if RE polypeptide affects cell viability, HFFF2 cells were cultured in the presence of RE for 24, 48, and 72 h. As shown in Figure 8, the viability of cells treated with RE or with H_2_O slightly decreased with respect to the viability of untreated cells. However, these differences are not significant. In particular, both RE and H_2_O induced a reduction of the percentage of living cells at the same extent, thus indicating that RE (300 μL/mL) did not induce a significant decrease of cell viability of HFFF2 cells at any of the analyzed time points (Figure 8).

#### 3.7.2. Evaluation of Dead and Living Cells by Trypan Blue Staining

**Figure 9:**
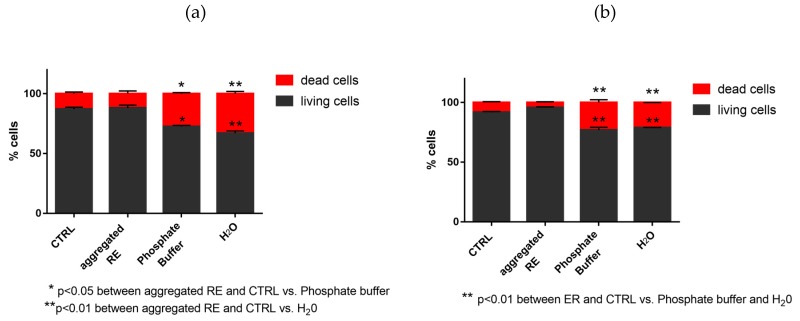
Percentage of living HFFF2 after 24 (**a**) and 72 h (**b**) from seeding.

Figure 9 shows that the percentage of living HFFF2 cells harvested from the aggregated RE-coated plates is significantly higher when compared with cells harvested from uncoated plates. In particular, at 24 and 72 h after seeding the cells, the percentage of living and dead cells was the same in RE-coated plates, as well as in control plates. On the contrary, the percentage of living cells in uncoated plates was significantly lower with respect to that observed in RE-coated plates.

## 4. Conclusions

The design and production of the chimeric RE polypeptide was successfully carried out. Herein, we have demonstrated that it is able to self-assemble in nanofibers under controlled conditions. The polypeptide ability to self-assemble was demonstrated by means of turbidimetric assays. The morphology of aggregates was analyzed by SEM and AFM microscopies. The molecular conformation was assessed by CD spectroscopy. The supramolecular structure of aggregated was compared with the lyophilized (not-aggregated) RE polypeptide. SR-induced XPS measurements allowed to successfully evaluate the molecular stability under aggregation condition of the polypeptide chains, and to hypothesize that the decrease of the intermolecular and intramolecular H-bonds is a consequence of the aggregation process. Previous studies on amyloidogenic elastin-like peptides have shown that H-bonds increased from lyophilized to aggregated samples. Herein, hydrogen-bond-induced effects on SR-XPS and NEXAFS spectra are observed mainly in the lyophilized samples. These findings suggest that the aggregation state is not of amyloid-like nature. NEXAFS N K-edge spectra fully confirmed these results. Finally, preliminary biological assays reported in this study showed the survival of human fibroblast cells on chimeric RE polypeptide. Overall, this work demonstrates that RE polypeptide is a promising candidate in the field of biomedical devices.

## Supplementary Materials

The following are available online at https://www.mdpi.com/2079-4991/9/11/1613/s1. Figure S1: DNA sequence. Figure S2: Western blotting. Figure S3: HPLC chromatogram. Figure S4: ESI–MS spectrum. Figure S5: Fiber width measurements on AFM images. Figure S6: SR-XPS O1s spectra. Table S1: Primers and PCR protocols. Table S2: Amino acid analysis. Table S3: C1s, O1s, and N1s SR-XPS data.
