# Supplementary material for: Fibrillar Self-Assembly of a Chimeric Elastin-Resilin Inspired Engineered Polypeptide
Source: Nanomaterials (Basel). 2019 Nov 14;9(11):1613. doi: 10.3390/nano9111613 (PMC6915571; doi:10.3390/nano9111613)
Supplement: Supplementary file 1 [file nanomaterials-09-01613-s001.pdf]

Fibrillar Self-Assembly of a Chimeric Elastin-Resilin Inspired Engineered Polypeptide

Angelo Bracalello [1], Valeria Secchi [2,*], Roberta Mastrantonio [2], Antonietta Pepe [1], Tiziana Persichini [2], Giovanna Iucci [2], Brigida Bochicchio [1,*] and Chiara Battocchio [2,*]

[1] Department of Sciences, University of Basilicata, Via Ateneo Lucano, 10, 85100 Potenza, Italy; angelo.bracalello@unibas.it (A.B.); antonietta.pepe@unibas.it (A.P.)
[2] Department of Sciences, University of Roma Tre, Via della Vasca Navale, 79, 00146 Rome, Italy; roberta.mastrantonio@uniroma3.it (R.M.); tiziana.persichini@uniroma3.it (T.P.); giovanna.iucci@uniroma3.it (G.I.)

* Correspondence: chiara.battocchio@uniroma3.it (C.B.); valeria.secchi@uniroma3.it (V.S.); brigida.bochicchio@unibas.it (B.B.); Tel: +39 06 5733 3400 (C.B.)

SUPPORTING INFORMATION

Gene *rel*

5'GGTAAAAAAAAGCCGGTATCGGATACCTATGGCGCTCCTGGCGGCGGTAATGGCGGGCGTCCGA
GCGATACATATGGTGCCCCGGGTGGAGGGAACGGCGGACGCCCGAGTGACACCTATGGCGCACCAG
GCGGAGGGAATGGCGGTCGCCCCTCCGATACGTACGGTGCGCCAGGTGGCGGCAACGGTGGGCGTC
CGGGCCTGGGCGGCGTAGGTCTGGGCGGTGTAGGTCTTGGTGGCGTTGGTAAG3′

Gene *eln*

5'GGTGCCCGTCCGGGTGTGGGTGTTGGCGGTATTCCGACCTATGGTGTGGGTGCCGGCGGTTTTC
CGGGTTTTGGTGTTGGTGTGGGCGGTATCCCGGGTGTTGCCGGCGTGCCGAGCGTTGGCGGTGTCC
CGGGCGTGGGCGGTGTTCCGGGTGTCGGCATTTCTAAACTGGGCGGTCTGGGTGTGGGCGGTCTGG
GTGTGCCGGGTGTTGGCGGTCTGGGCGGTCGTGGCGATAGCCCGCTGGGCGGTCTGGGCGTTGGCG
GTCTGGGCGTTCCTGGTGTGGGCGGTTTAGGCAAAGGTGCACGTCCGGGTGTTGGTGTCGGCGGTA
TTCCGACGTACGGTGTGGGCGCTGGCGGTTTTCCGGGTTTCGGCGTTGGCGTTGGCGGTATTCCGG
GTGTTGCCGGTGTCCCGAGTGTGGGCGGTGTTCCTGGTGTCGGCGGTGTGCCGGGCGTTGGTATCT
CCGGTAAGCCTGGT3'

Figure S1. The designed oligonucleotide sequences of the (A) rel, and (B) eln genes. rel gene codifies for K-Res-$(LGGVG)_3$-K and eln gene codifies for EX20-K-EX30_18-RGD-EX30_18-K-EX20-K.

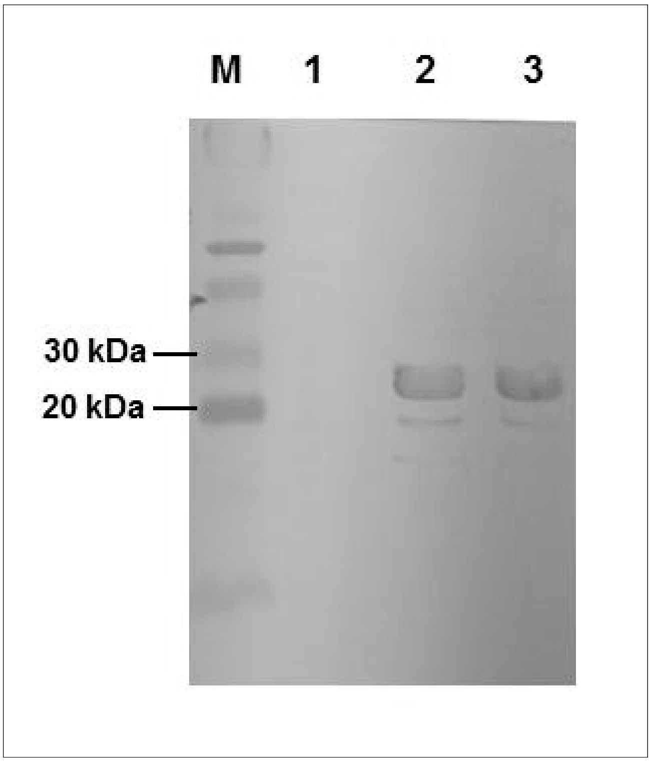

Figure 2. Western blotting analysis of His_6 RE polypeptide in induced and uninduced BL21DE3 cells. Total cell lysates were size fractionated in a 12%-4% SDS-PAGE gel. The fractioned proteins were transferred to a PVDF membrane and the RE polypeptide was detected by incubation with monoclonal anti-polyhistidine peroxidase conjugate antibody (Sigma). Lane M: ColorBurst™ electrophoresis marker (8,000-220,000 Da, Sigma); lane 1: uninduced culture; lane 2,3: induced culture.

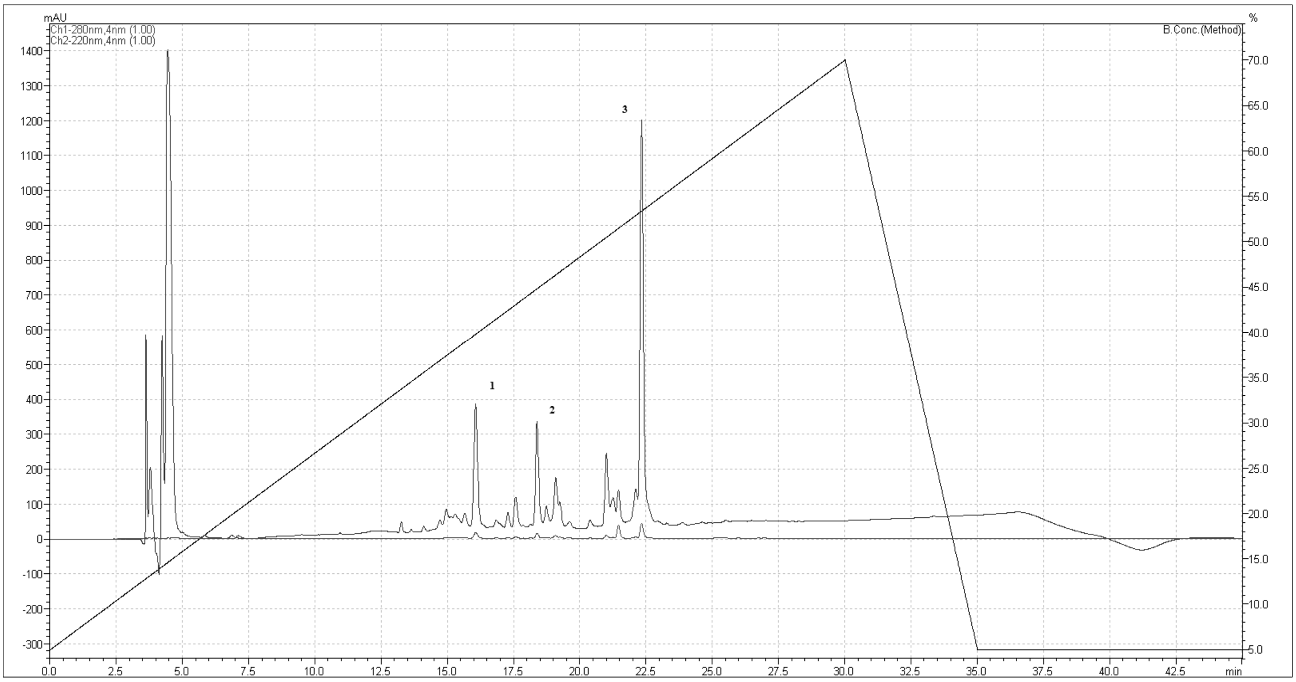

Figure S3. RP-HPLC chromatogram of RE polypeptide. Three fractions were collected at the following retention time 16.2 (peak 1), 18.6 (peak 2) and 22.4 min (peak 3).

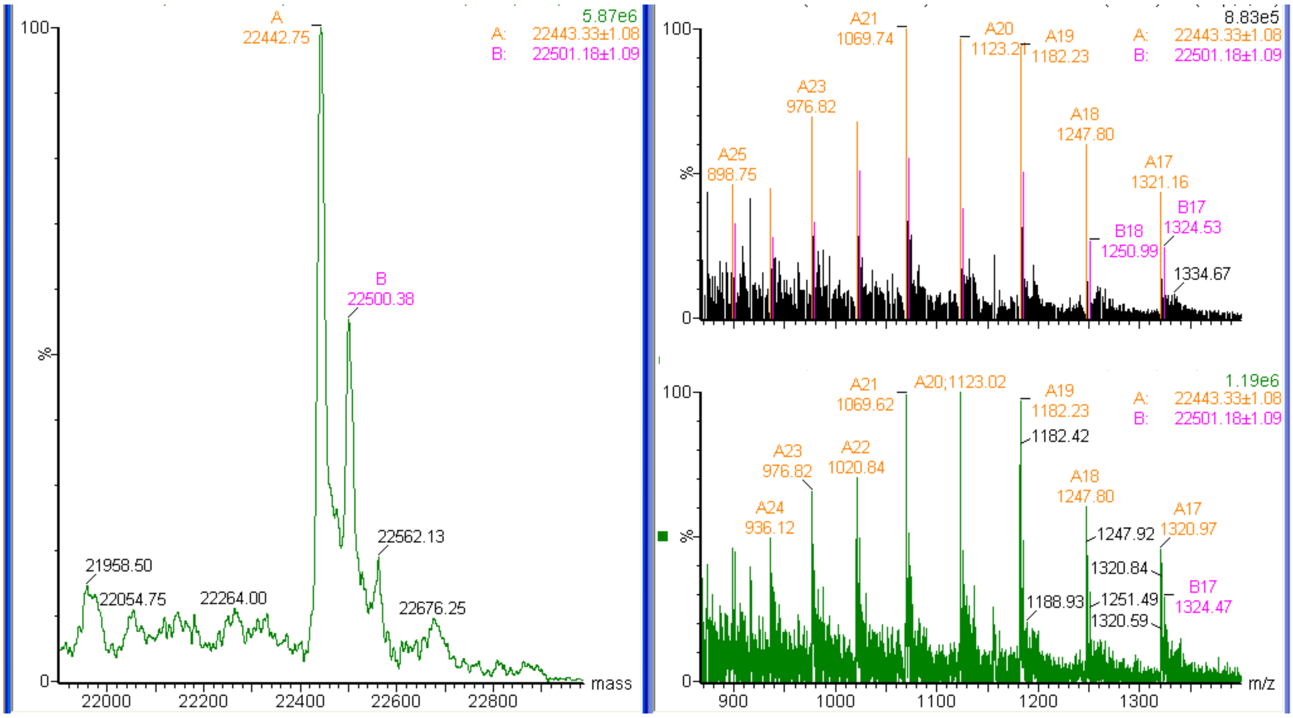

Figure S4. ESI-MS spectrum of RE polypeptide: deconvoluted mass spectrum (left); experimental ESI-MS spectra showing multiply charged ions of RE polypeptide (right). An label corresponds to $[M+nH]^{n+}$, while Bn label corresponds to$[M+(n-2)H+Na+K]^{n+}$.

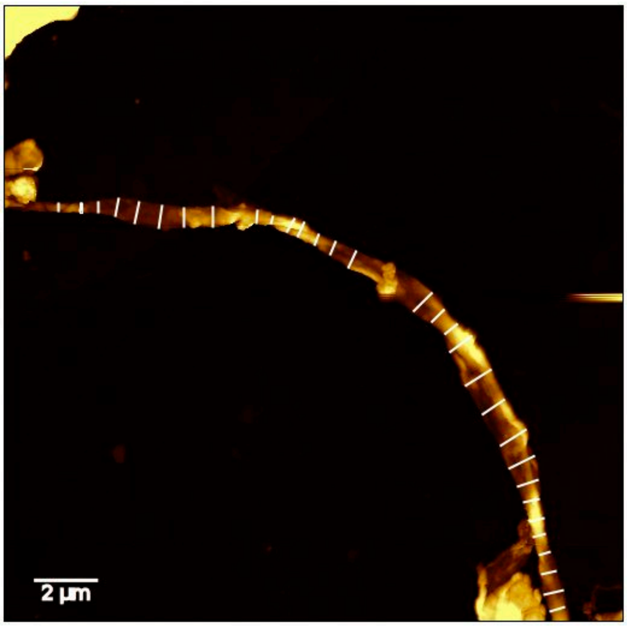

Figure S5. AFM image of aggregated RE polypeptide. The white lines highlight the diameter measurements performed by ImageJ measurement tool. The mean diameter of the fiber is 0.588 ± 0.210 m (n=33), ranging from a minimum value of 0.283 m to a maximum measured value of 1.005 m.

Table S1: Details on utilized PCR protocols

**Primers**	**Sequences** [a]	**PCR cycles**[b]
rel* forward**<br>rel* reverse**	5-'GGTAAAAAAAAGCCGGTATCG-3'<br>5-'GAAGACGAACCAAGCTTACCAACGCCA-3'	95 °C x 5';<br>35 cycles: 95 °C x 1', 56 °C x1', 70 °C x 1';<br>70 °C x 5'.
eln* forward**<br>eln* reverse**	5-'GAAGACGA**T**GGTGCCCGTCCGGGTGT-3'<br>5-'GAAGACGAACCAGGCTTACCGGAGATAC-3'	95 °C x 5';<br>35 cycles: 95 °C x 1', 58 °C x 1', 70 °C x 1';<br>70 °C x 5'.
re* forward**<br>re* reverse**	5'-GACGACGACAAGATGGGTAAAAAAAAGCCGGTATCG-3'<br>5'-GAGGAGAAGCCCGGTTAACCAGGCTTACCGGAGATAC-3'	95° C x 5';<br>30 cycles: 95°C x 1', 60°C x 1', 70 °C x 1'; 70 °C x 5'

[a] BbsI restriction site is underlined; the T (in bold) downstream of the BbsI restriction site, is necessary to restore the correct nucleotide sequence of the chimeric gene;
[b] PCR reaction utilized Eurotaq polymerase (EuroClone), dNTP (Amersham) in a Thermal Cycler peqSTART96 (Sigma);

Table S2. Experimental and theoretical results of amino acid composition of RE

	**Molar ratio**			**Molar ratio**	
	Experimental	Theoretical		Experimental	Theoretical
Asx	13.9	13	Ile	5.88	6
Thr	5.88	6	Leu	11.95	12
Ser	8.25	9	Tyr	5.88	6
Glx	0.29	-	Phe	4.08	4
Pro	26.37	27	His[a]	8.73	6
Gly	106.34	105	Lys	7.97	8
Ala	11.00	11	Arg	7.30	7
Val	34.91	35	Cys	n.d.	-
Met	n.d.	2	Trp	n.d.	-

[a] His quantification is affected by the high peak of Gly that elutes near His retention time

Table S3. *C1s, O1s, N1s SR-XPS data*

Table S3. C1s, O1s, N1s SR-XPS data				
**C1s**	**Center**	**Area**	**FWHM**	Assignment
RE				
	285.00	58551.8	1.46	C-C
	286.48	13565	1.46	C-N;C-O
	288.45	8525.1	1.46	N-C=O---H
Aggregated RE				
	285.00	1869.9	1.4	C-C
	286.55	845.28	1.4	C-N;C-O
	287.786	598.834	1.4	N-C=O
**O1s**	Center	Area	FWHM	Assignment
RE				
	531.56	1620	1.78	N-C=O
	532.52	1893	1.78	O-C
	534.06	145	1.78	Phys. H_2O
Aggregated RE				
	531.60	4606	1.26	N-C=O
	534.36	854	1.26	O-C
	535.50	133	1.26	Phys. H_2O
**N1s**	Center	Area	FWHM	Assignment
RE				
	399.24	435	2.17	
	400.78	128	2.17	
Aggregated RE				
	398.86	345	1.57	
	400.50	53	1.57	
